# Extensive diet-induced atherosclerosis in scavenger receptor class B type 1-deficient mice is associated with substantial leukocytosis and elevated vascular cell adhesion molecule-1 expression in coronary artery endothelium

**DOI:** 10.3389/fphys.2022.1023397

**Published:** 2023-01-12

**Authors:** Mark T. Fuller, Omid Dadoo, Ting Xiong, Pardh Chivukula, Melissa E. MacDonald, Samuel K. Lee, Richard C. Austin, Suleiman A. Igdoura, Bernardo L. Trigatti

**Affiliations:** ^1^ Department of Biochemistry and Biomedical Sciences, McMaster University, Hamilton, ON, Canada; ^2^ Thrombosis and Atherosclerosis Research Institute, Hamilton Health Sciences and McMaster University, Hamilton, ON, Canada; ^3^ Department of Medicine, Division of Nephrology, The Research Institute of St. Joe’s Hamilton and the Hamilton Center for Kidney Research, McMaster University, Hamilton, ON, Canada; ^4^ Department of Biology and Department of Pathology and Molecular Medicine, McMaster University, Hamilton, ON, Canada

**Keywords:** atherosclerosis, cholesterol, coronary artery, HDL, inflammation, knockout, lipoprotein, receptor

## Abstract

High levels of low density lipoprotein (LDL) cholesterol and low levels of high density lipoprotein (HDL) cholesterol are risk factors for cardiovascular disease. Mice that lack genes involved in the clearance of LDL from the bloodstream, such as the LDL receptor and apolipoprotein E, are widely used models of experimental atherosclerosis. Conversely, mice that lack the HDL receptor, scavenger receptor class B type I, and therefore have disrupted HDL functionality, also develop diet-inducible atherosclerosis but are a seldom-used disease model. In this study, we compared atherosclerosis and associated phenotypes in scavenger receptor class B type I knockout mice with those of wild type, LDL receptor knockout, and apolipoprotein E knockout mice after 20 weeks of being fed an atherogenic diet containing sodium cholate. We found that while scavenger receptor class B type I knockout mice had substantially lower plasma cholesterol than LDL receptor and apolipoprotein E knockout mice, they developed atherosclerotic plaques with similar sizes and compositions in their aortic sinuses, and more extensive atherosclerosis in their descending aortas and coronary arteries. This was associated with elevated tumor necrosis factor alpha levels in scavenger receptor class B type I knockout mice compared to wild type and LDL receptor knockout mice, and lymphocytosis, monocytosis, and elevated vascular cell adhesion molecule expression in coronary artery endothelial cells compared to the other mice examined. We conclude that extensive atherosclerosis in arteries that are not generally susceptible to atherosclerosis in scavenger receptor class B type I knockout mice is driven by factors in addition to hypercholesterolemia, including inflammation, dysregulation of the immune system and increased sensitivity of endothelial cells in arteries that are normally resistant to atherosclerosis. Scavenger receptor class B type I knockout mice fed a cholate containing atherogenic diet may prove to be a useful model to study mechanisms of atherosclerosis and evaluate treatments that rely on intact LDL clearance pathways.

## Introduction

The incidence of atherosclerosis has been established to positively correlate with the ratio of the low density lipoprotein (LDL) cholesterol to high density lipoprotein (HDL) cholesterol concentration in plasma (Fernandez and Webb, 2008). Atherosclerosis is an inflammatory disease characterized by the build-up of cholesterol rich plaque in the walls of affected arteries and is the leading cause of cardiovascular disease globally (Libby, 2002; Libby, 2021; Soehnlein and Libby, 2021). Atherosclerotic plaque development is initiated at sites along the vasculature where the endothelium is disrupted by non-laminar blood flow (Chatzizisis et al., 2007). At these sites, the endothelium is more permeable to components of the blood such as LDL particles (Chatzizisis et al., 2007). Moreover, the endothelial cells in these regions tend to express high levels of vascular cell adhesion molecule 1 (VCAM-1) (Iiyama et al., 1999), which creates favourable sites for the attachment of circulating monocytes (Chatzizisis et al., 2007). Monocytes give rise to macrophages, which phagocytose trapped LDL particles that have been oxidatively modified, resulting in the formation of cholesterol engorged macrophage foam cells (Libby, 2002), the major cell type that characterizes early stage atherosclerotic lesions (Yu et al., 2013).

While high levels of LDL in the blood stream drive atherosclerosis, HDL levels are inversely correlated with cardiovascular disease (Castelli, 1988). This is thought to be in large part due to their role in facilitating cholesterol efflux from peripheral tissues and subsequent transport to the liver for excretion, a process called reverse cholesterol transport (Linsel-Nitschke and Tall, 2005). In addition to their role in reverse cholesterol transport, HDLs have been shown to suppress expression of VCAM-1 on endothelial cells (Kimura et al., 2006), and induce the migration of macrophages (Al-Jarallah et al., 2014), which may contribute to the regression of atherosclerotic plaques (Llodrá et al., 2004). The scavenger receptor class b type 1 (SR-B1) is an HDL receptor that is required in all of these functions.

Genetic disruptions of lipoprotein metabolism through targeted knockout (KO) of the LDL receptor (LDLR) (Ishibashi et al., 1993) or of apolipoprotein E (ApoE) (Piedrahita et al., 1992), a major ligand for lipoprotein receptors, has given rise to the conventional mouse models of atherosclerosis that are widely used in basic atherosclerosis research today (Whitman, 2004). These mice have elevated LDL- and VLDL (very low density lipoprotein)-cholesterol, and develop atherosclerotic lesions either spontaneously (ApoE KO) or when fed high fat/high cholesterol diets (LDLR KO) in major arteries such as the aortic sinus, aortic arch and branch points along the descending aorta; sites where blood flow is non-laminar (Whitman, 2004). When SR-B1 is additionally knocked out in ApoE-mutant or LDLR KO mice, the resulting double KO mice are uniquely susceptible to development of occlusive coronary artery atherosclerosis and myocardial infarction causing early death (Braun et al., 2002; Zhang et al., 2005; Fuller et al., 2014). Interestingly, diet induced coronary artery atherosclerosis in SR-B1/LDLR dKO mice is correlated with increased expression of VCAM-1 in coronary arteries, elevated monocyte and lymphocyte counts in blood, and high levels of inflammatory cytokines in plasma compared to LDLR KO mice that are fed the same diets (Fuller et al., 2014). This severe phenotype occurs despite a reduction in plasma VLDL and LDL cholesterol (Fuller et al., 2014), suggesting that whole body SR-B1 deficiency affects multiple drivers of atherosclerosis independently of VLDL and LDL cholesterol.

Few studies have used atherogenic diet fed SR-B1 single KO mice as animal models to study atherosclerosis (Van Eck et al., 2003; Huby et al., 2006; Harder et al., 2007; Hildebrand et al., 2010). While long-term feeding of a Western type diet (Van Eck et al., 2003; Hildebrand et al., 2010) or a high fat, high cholesterol diet (Harder et al., 2007) leads to the development of small atherosclerotic plaques in the aortic sinus in SR-B1 KO mice; a high fat, high cholesterol diet containing sodium cholate (HFCC diet) generates large plaques in the aortic sinus after 11 weeks (Huby et al., 2006). However, the extent of atherosclerosis in the aortic arches, descending aortas or coronary arteries has not been reported. In this study, we examined the effects of HFCC diet feeding on atherosclerosis development in SR-B1 KO mice in comparison to wild type (WT), LDLR KO, and ApoE KO mice. We demonstrate that atherosclerotic plaque size in the aortic sinus of SR-B1 KO mice after 20 weeks of HFCC is similar to that of LDLR KO and ApoE KO mice, while atherosclerosis in the descending aorta and coronary arteries is significantly higher in SR-B1 KO mice. This corresponds with higher VCAM-1 expression in coronary arteries of SR-B1 KO mice, despite substantially lower plasma cholesterol levels. We conclude that HFCC diet fed SR-B1 KO mice are a model of wide-spread experimental atherosclerosis.

## Materials and methods

### Animals and diet

All experiments involving mice were approved by the McMaster University Animal Research Ethics Board and were carried out following guidelines set by the Canadian Council on Animal Care. C57BL6/J wild type (WT) mice (RRID:IMSR_JAX:000664) and LDLR KO (RRID:IMSR_JAX:002207) and ApoE KO mice (RRID:IMSR_JAX:002052) on C57BL6/J backgrounds were originally purchased from Jackson Labs, and bred in house. SR-B1 KO mice were originally obtained from M. Krieger at the Massachusetts Institute of Technology and backcrossed >10 times onto a C57BL6/J background in house. To generate SR-B1 KO mice for experiments, male and female SR-B1^KO/KO^ breeders were fed a diet consisting of Prolab RMH3000 containing 0.5% probucol (Lab Diet, St. Louis MO, United States of America). Pups were placed on normal diet (Teklad TD 2018; Envigo, Madison WI) at weaning. Female mice were used for the majority of the experiments in this study. All mice were maintained on a 12 h light/dark cycle in vented cages with automated watering and free access to food.

Normal diet (6.2% fat; 0% cholesterol; Teklad TD 2018; Envigo, Madison WI) fed mice (10 weeks old) were fasted, anesthetized with isoflurane (3% in O_2_) and heparinized blood was collected from tail veins. Mice were allowed to recover and were then fed a diet (Teklad TD.88051, Envigo, Madison WI) consisting of 15.8% fat (7.5% from cocoa butter), 1.25% cholesterol and 0.5% sodium cholate (HFCC diet) for 20 weeks. After HFCC diet feeding, mice were fasted for 4 h, euthanized by thoracotomy under complete anesthesia and heparinized blood (cardiac puncture) and tissues were collected for analysis. A separate cohort of female SR-B1^KO/KO^ and WT mice were fed the HFCC diet for 12 weeks beginning at 14 weeks of age, and were fasted for 14 h prior to blood collection for corticosterone analysis.

### Plasma lipids

Plasma was prepared by microcentrifugation from heparinized blood. Total cholesterol (Cholesterol Infinity, Thermo Fisher Scientific, Ottawa, ON, Canada), unesterified cholesterol (Free Cholesterol E, WAKO Diagnostics, Mountain View, CA, United States), HDL cholesterol (HDL Cholesterol E, WAKO Diagnostics, Mountain View, CA, United States) and Triglyceride (L-Type Triglyceride M, WAKO Chemicals, Richmond, VA, United States) were measured using the indicated commercial assay kits and following manufacturers’ instructions. The HDL Cholesterol E kit uses phosphotungstate-magnesium to precipitate lipoproteins other than normal HDL (Durrington, 1980). Therefore, the cholesterol detected after precipitation is referred to as (non-precipitable) HDL cholesterol. Non-HDL cholesterol was calculated as total cholesterol—(non-precipitable) HDL cholesterol.

### Atherosclerosis analysis

For analysis of atherosclerosis in the aortic sinus and coronary arteries, hearts were excised, fixed overnight in 10% formalin, and prepared and sectioned as described previously (Covey et al., 2003; Fuller et al., 2014). 10μm-thick transverse cryosections were collected from the middle of the heart to the base of the aortic sinus in 0.5 mm intervals, then in 100 μm intervals to the top of the valve leaflets. Atherosclerotic plaque was detected by oil red-O staining; nuclei were visualized with Meyer’s hematoxylin stain. ImageJ software was used to measure the cross-sectional area of atherosclerotic plaque in the section best represented by 3 intact valve leaflets. Atherosclerosis in coronary arteries was evaluated by counting the coronary arteries in at least 3 cryosections that contained raised plaque which occluded all, or a portion of the lumen of the artery.

Atherosclerosis from *en face* prepared-aortas was assessed as previously described (Covey et al., 2003). Briefly, aortas were excised, fixed in 10% formalin, cleaned and stained with Sudan-IV. Aortas were opened longitudinally and mounted on a glass slide with aqueous mounting medium and a cover slip. Aortas were imaged with a Nikon digital SLR camera, and plaque coverage as a percentage of total vessel area was measured manually using ImageJ software. Anatomical boundaries were used to define the aortic arch, the thoracic aorta and the abdominal aorta. The arch was defined as the region from the top of the heart to the end of the lesser curvature. The thoracic aorta was defined as the region from the aortic arch to the attachment point of the diaphragm. The abdominal aorta was defined as the region from the diaphragm to the iliac bifurcation.

### Histological characterization of atherosclerotic plaques

Necrotic cores in aortic sinus atherosclerotic plaques were defined as regions of the plaque that contained no nuclei in hematoxylin and eosin (H&E) stained sections, and were measured manually using ImageJ software. Collagen was detected as blue-stained material in sections stained with Masson’s trichrome (Sigma, Oakville, ON, Canada) and quantified using the colour deconvolution and threshold functions in ImageJ software. CD68 and smooth muscle actin (SMA) were detected by immunofluorescence using rat anti-mouse CD68 (FA11, AbD Serotec, Cat # MCA 1957, Bio-Rad Laboratories, CA, United States; RRID:AB_322219) and rabbit anti-mouse SMA (Abcam Inc, Cat # ab5694, Cambridge MA United States; RRID:AB_2223021) primary antibodies and Alexa 594 goat anti-rat IgG (H + L) (Cat #A-11007; RRID:AB_10561522), and Alexa 488 goat anti-rabbit IgG (H + L) (Cat # A-11008; RRID:AB_143165) secondary antibodies (Life Technologies Inc, Burlington, Ontario Canada), respectively. Nuclei were counterstained with 4′,6-Diamidino-2-phenylindole dihydrochloride (DAPI). CD68-and SMA-positive areas were measured using the threshold function in ImageJ software.

### Blood cell analysis

Blood was collected from the tail veins of mice after 6 weeks of HFCC feeding. Fresh, heparin anti-coagulated blood was run on a Hemavet Multi-Species Hematology System as described previously (Fuller et al., 2014). Ly6C expression was assessed by flow cytometry of whole blood as described previously (Fuller et al., 2014). CD11b and CD115 double-positive monocytes were identified then analyzed for Ly6C expression using FITC-, PE- and APC-conjugated antibodies (BD Biosciences, San Jose CA, United States), respectively.

### Cytokine and corticosterone analysis

Interleukin 6 (IL-6) and Tumor Necrosis Factor alpha (TNF-α were measured in plasma after 20 weeks of HFCC feeding. IL-6 and TNF-α were measured using commercial ELISA kits (BioLegend, San Diego, CA, United States) following manufacturer’s protocols. Corticosterone levels were analyzed in plasma prepared from mice fasted for 14 h using a competitive ELISA kit (Invitrogen Cat. #EIACORT; Thermo Fisher Scientific, Ottawa, ON, Canada).

### VCAM-1 immunofluorescence

VCAM-1 was detected by immunofluorescence as described previously (Fuller et al., 2014), using cell culture supernatants from rat B-lymphocyte hybridoma cells (P3C4) that produce anti-mouse VCAM-1 antibody, and Alexa 594 goat anti-rat secondary antibody (Life Technologies Inc, Burlington, Ontario Canada). The P3C4 hybridoma developed by E.A. Wayner and T. LeBien (Fred Hutchinson Cancer Research Center) was obtained from the Developmental Studies Hybridoma Bank, created by the NICHD of the NIH and maintained at The University of Iowa, Department of Biology, Iowa City, IA 52242. The artery wall auto fluoresces green and nuclei were detected with DAPI counterstain.

### Statistical analysis

GraphPad Prism software (version 9.4.1) was used for statistical analysis. Data sets consisting of 3 or more groups with two variables were analyzed by two-way ANOVA with Tukey’s Multiple Comparisons Post-Hoc test. Data sets consisting of 3 or more groups with one variable were analyzed by one-way ANOVA with Tukey’s Multiple Comparisons Post-Hoc test. Data sets consisting of 2 groups were analyzed using the Mann-Whitney Rank Sum test. Differences were considered statistically significant if *p* < 0.05.

## Results

### Plasma lipids

**FIGURE 1:**
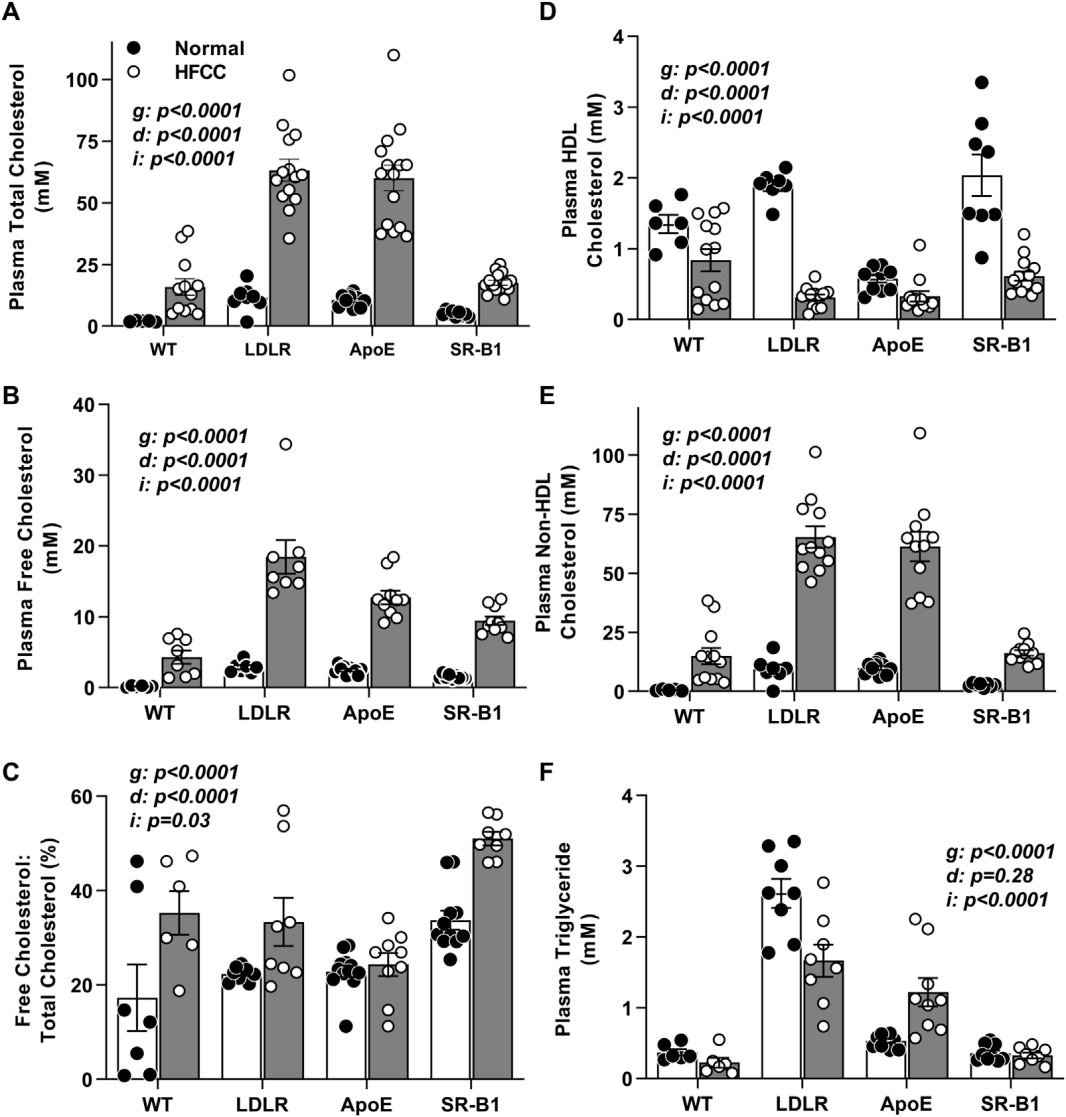
Plasma lipid parameters in HFCC-diet fed female WT, LDLR KO, ApoE KO, and SR-B1 KO mice. Blood was collected after fasting from 10-week old female mice fed a normal diet (black symbols) or from mice fed the HFCC diet for 20 weeks beginning at 10 weeks of age (white symbols). Bars represent average plasma lipid levels and symbols represent data from individual WT (*N* = 6–13), LDLR KO (*N* = 8–14), ApoE KO (*N* = 9–15), and SR-B1 KO (*N* = 8–15) mice for **(A)** total cholesterol, **(B)** unesterified (free) cholesterol, **(C)** free cholesterol: total cholesterol ratio, **(D)** HDL cholesterol, **(E)** non-HDL cholesterol, and **(F)** triglyceride. Error bars represent standard errors of the mean. Statistical significance was analyzed by two way ANOVA. *p* values are indicated for the genotype (*g*) and diet (*d*) variables and the interaction (*i*) between the effects of genotype and diet.

Mice were fed the HFCC diet beginning at an age (10 weeks) preceding the appearance of atherosclerotic plaques even in ApoE KO mice (Veerman et al., 2013). We first examined plasma lipids in mice before and after the HFCC diet feeding period (Figure 1). Plasma total cholesterol levels were elevated by HFCC diet feeding in all 4 groups, reaching the highest levels in HFCC diet-fed LDLR KO and ApoE KO mice (Figure 1A). Plasma total cholesterol levels in chow-fed SR-B1 KO mice were approximately 2-fold higher than those in wild type mice but were elevated to similar levels as those in wild type mice after HFCC diet feeding. HFCC diet feeding also resulted in increased plasma free cholesterol levels in all strains of mice, with the highest levels being reached LDLR mice (Figure 1B). However SR-B1 KO mice fed the HFCC diet exhibited the highest plasma unesterified:total cholesterol ratios (approximately 50%; Figure 1C), consistent with previous reports of the accumulation of unesterified cholesterol in lipoproteins in SR-B1 KO mice (Braun et al., 2003; Dole et al., 2008; Hildebrand et al., 2010; Korporaal et al., 2011). HDL cholesterol (non-precipitable) levels were highest in SR-B1 KO and lowest in ApoE KO mice fed normal chow. However HFCC diet feeding resulted in decreases in the amount of detectable HDL cholesterol in all strains including SR-B1 KO mice (Figure 1D), and corresponding increases in non-HDL cholesterol (Figure 1E), which mirrored levels of total cholesterol (Figure 1A). Non-HDL cholesterol levels in both chow and HFCC diet fed mice were significantly higher in LDLR KO and ApoE KO mice than in WT or SR-B1 KO mice (Figure 1E). Triglycerides were significantly higher in chow fed LDLR KO mice and were reduced after HFCC diet feeding, whereas they were elevated by HFCC diet feeding in ApoE KO mice and not altered significantly by HFCC diet feeding in wild type and SR-B1 KO mice (Figure 1F).

### Aortic sinus atherosclerosis and plaque composition

**FIGURE 2:**
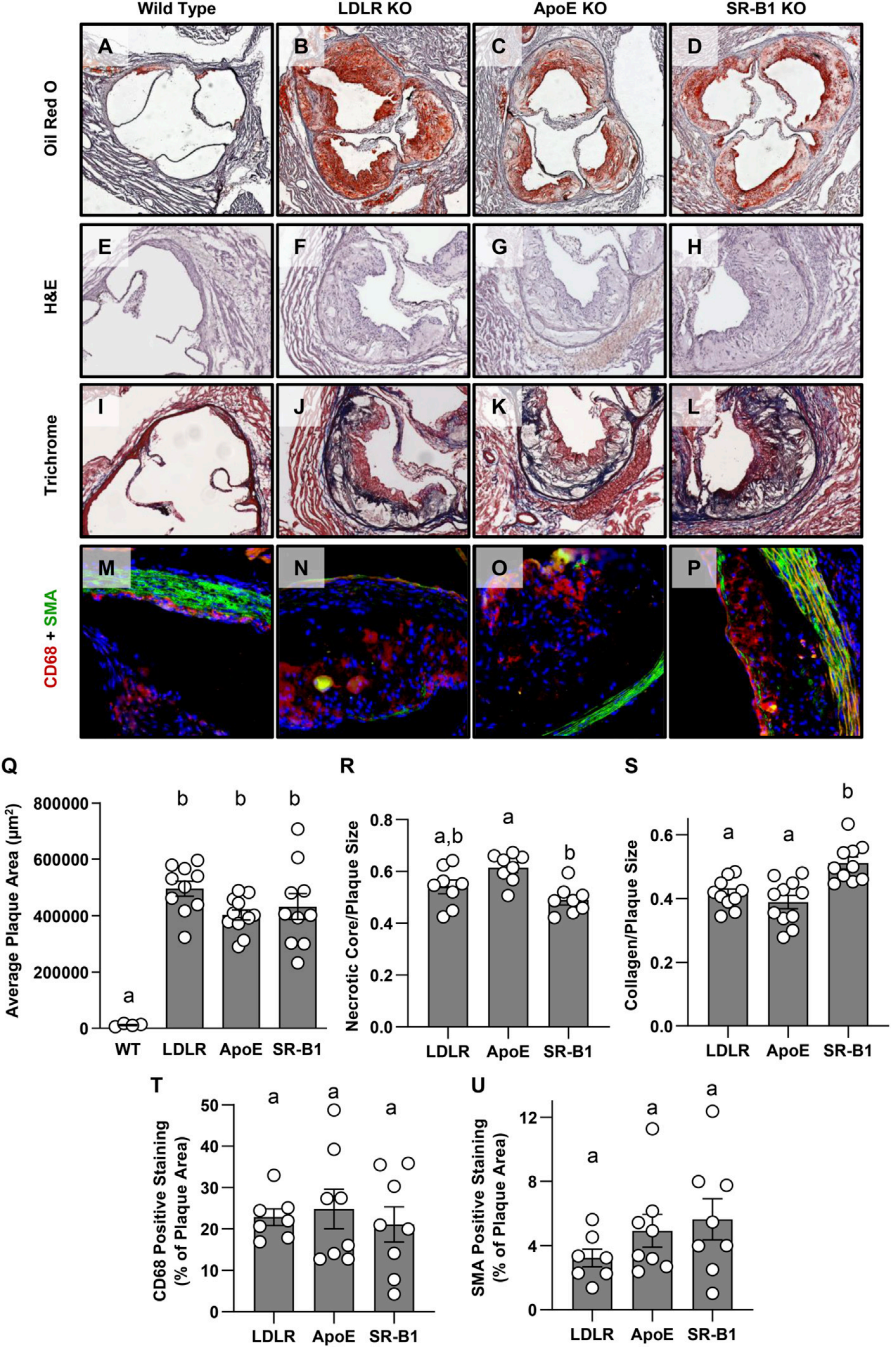
Size and composition of aortic sinus atherosclerotic plaques in HFCC-fed female WT, LDLR KO, ApoE KO, and SR-B1 KO mice. Representative images of a series of transverse cryosections of an aortic sinus from one mouse of the indicated genotype stained with oil red-O **(A–D)**, H&E **(E–H)**, Masson’s trichrome **(I–L)** or by immunofluorescence for CD68 (red) and smooth muscle actin (green) **(M–P)** are shown. **(Q)** Atherosclerotic plaque cross sectional areas at the aortic root. **(R)** Necrotic core size as a fraction of plaque size measured as acellular regions in H&E stained sections. **(S)** Collagen as a fraction of plaque size measured as blue-stained tissue in Masson’s trichrome-stained sections. **(T)** CD68-positive area as a fraction of plaque size as measured by immunofluorescence. **(U)** SMA-positive area as a fraction of plaque size as measured by immunofluorescence. Individual symbols represent data from individual mice and bars with error bars represent average ± SEM of each group. Only plaque size was measured in WT mice due to difficulty reproducibly identifying plaques in images stained for other parameters. Bars with different letters are statistically significantly different from one another by one-way ANOVA with Tukey’s post-hoc test. *p* values are **(Q)**: Overall ANOVA: *p* < 0.0001; multiple comparisons: *p* < 0.0001 for a vs. b; **(R)**: Overall ANOVA: *p* = 0.003; multiple comparisons: *p* = 0.0019 for a vs. b; **(S)**: Overall ANOVA: *p* = 0.0002; multiple comparisons: *p* < 0.005 for a vs. b; **(T)**: Overall ANOVA and multiple comparisons: *p* > 0.7 (not significant); **(U)**: Overall ANOVA and multiple comparisons: *p* > 0.25 (not significant).

Atherosclerotic plaques in aortic sinuses of female WT, LDLR KO, ApoE KO, and SR-B1 KO mice after 20 weeks of HFCC feeding were stained with oil red O and hematoxylin (Figures 2A–D). Atherosclerotic plaque development was minimal in WT mice, while extensive atherosclerosis was observed in the aortic sinuses of each of the three KO groups. In female mice, there were no significant differences in average plaque areas among the three KO groups (Figure 2Q). However, in male mice, plaques from SR-B1 KO mice were significantly smaller than plaques from LDLR KO and ApoE KO mice, but significantly larger than those of WT mice (Supplementary Figure S1). Figures 2E–H show representative hematoxylin and eosin stained aortic sinus plaques from each of the four groups of female mice. Necrotic cores were identified as regions of the plaque that contained no nuclei. No necrotic cores were observed in WT plaques, while large necrotic cores were observed in the plaques from SR-B1 KO as well as LDLR KO and ApoE KO mice. Normalized to plaque size, necrotic cores in plaques from ApoE KO mice were statistically significantly larger than in plaques from SR-B1 KO mice, but not significantly different from those of LDLR KO mice (Figure 2R). Necrotic core size was not significantly different between SR-B1 KO and LDLR KO mice (Figure 2R). Masson’s trichrome staining was performed to reveal collagen deposition (Figures 2I–L: Collagen-rich fibrous regions of plaque stain blue). Plaques from SR-B1 KO mice contained the highest amount of collagen as a proportion of plaque size, with a slightly but significantly greater ratio of collagen:plaque area compared to both LDLR KO and ApoE KO plaques (Figure 2S). Plaques from LDLR KO and ApoE KO mice were not significantly different in collagen content (Figure 2S). Macrophages and smooth muscle cells were detected by immunostaining for CD68 and smooth muscle actin (SMA) (Figures 2F–M) and did not differ in plaques from LDLR KO, ApoE KO and SR-B1 KO mice did (Figures 2T, U). Most plaques from all KO groups displayed SMA-positive staining near the luminal surface of the plaque, suggesting smooth muscle cell infiltration and formation of fibrous caps. Because atherosclerotic plaques were either absent or very small in the aortic sinuses of WT mice (Figures 2A, E, I, M) quantification of necrotic core sizes or collagen, CD68 or SMA content was not carried out in that group.

### Atherosclerosis in the descending aorta and coronary arteries

**FIGURE 3:**
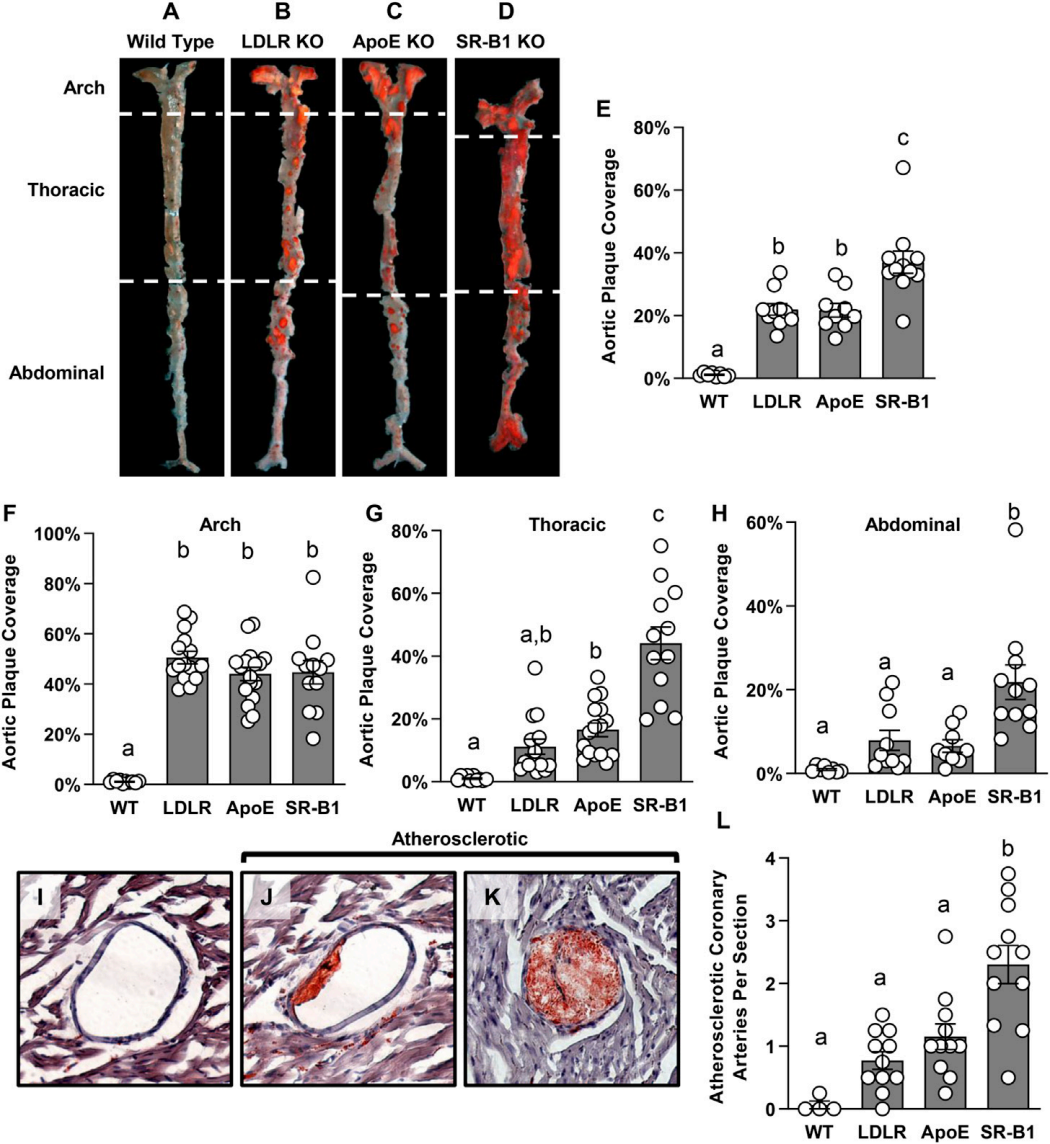
Atherosclerosis in the descending aorta and coronary arteries of HFCC-fed female WT, LDLR KO, ApoE KO and SR-B1 KO mice. **(A–D)** Representative en-face images of Sudan-IV-stained whole aortas from mice in the indicated genotype group. Dashed white lines indicate the boundaries of regions defined as the aortic arch, thoracic aorta and abdominal aorta. **(E–H)** Plaque coverage as a percentage of entire en-face vessel area in the whole aorta **(E)** (*N* = 8–11 per group), the aortic arch **(F)** (*N* = 10–17 per group), the thoracic aorta **(G)** (*N* = 10–16 per group) and the abdominal aorta **(H)** (*N* = 8–11 per group). **(I–K)** Representative images of oil red-O-stained coronary arteries that are plaque free **(I)** or partially **(J)** or fully **(K)** occluded with atherosclerotic plaque. All coronary artery cross sections containing atherosclerotic plaques were counted in at least 3 transverse myocardial cross-sections spaced at least 0.5 mm apart from each mouse. The graph in **(L)** represents the average number of atherosclerotic coronary artery cross sections observed per myocardial cross-section (*N* = 4 (WT) or 11 (LDLR KO, ApoE KO and SR-B1 KO). Individual symbols represent data from individual mice and bars with error bars represent average ± SEM of each group. The different letters indicate groups that are statistically significantly different from one another by one-way ANOVA with Tukey’s post-hoc test. **(E)** Overall ANOVA: *p* < 0.0001; multiple comparisons: *p* < 0.0001 for a vs*.* b or c; **(F)** Overall ANOVA: *p* < 0.0001; multiple comparisons: *p* < 0.0001 for a vs. b; *p* < 0.006 for b vs. c; **(G)** Overall ANOVA: *p* < 0.0001; multiple comparisons: *p* = 0.005 for a vs. b; *p* < 0.0001 for c vs. a or b; **(H)** Overall ANOVA: *p* < 0.0001; multiple comparisons: *p* < 0.005 for a vs. b; **(L)** Overall ANOVA: *p* < 0.0001; multiple comparisons: *p* < 0.004 for a vs. b.

We measured atherosclerosis in other arteries to determine if there is a difference in the distribution of plaque in the different strains of mice examined here. Atherosclerosis in descending aorta was measured *en face* by Sudan IV staining (Figures 3A–H). Surprisingly, there was a significant ∼2-fold greater level of atherosclerotic plaque coverage in the descending aortas of SR-B1 KO mice compared to those of LDLR KO and ApoE KO mice, which were not significantly different from each other (Figures 3A–E). All three KO groups developed significantly more aortic plaque than WT control groups, which developed very little aortic atherosclerosis. For a more detailed analysis of plaque development along the descending aorta, we divided the aorta into three regions: The arch, the thoracic aorta (from the end of the arch to the diaphragm) and the abdominal aorta (from the diaphragm to the iliac bifurcation). In both ApoE and LDLR KO mice, atherosclerotic plaque coverage (40%) was highest in the arch (Figure 3F) but dropped off significantly in the thoracic (15%–20% coverage, Figure 3G) and the abdominal (<10% coverage, Figure 3H) regions of the aorta. Likewise, plaque coverage in the aortic arch of SR-B1 KO mice averaged 40% (Figure 3G), similar to that in ApoE and LDLR KO mice. However, in contrast, plaque coverage did not drop off in the SR-B1 KO mice in the thoracic aorta (∼40%, Figure 3G) and decreased (<20%) only in the abdominal regions (Figure 3H) of aortas, extending to the iliac bifurcation, which was generally atherosclerosis free in the LDLR and ApoE KO mice. Therefore SR-B1 deficiency, even in otherwise WT mice, appears to cause regions of the aorta that are typically relatively atherosclerosis resistant to become more susceptible to HFCC diet induced atherosclerosis development.

Since SR-B1 deficiency renders other atherosclerotic mouse strains susceptible to coronary artery atherosclerosis (Braun et al., 2002; Zhang et al., 2005; Fuller et al., 2014), we analyzed atherosclerosis in coronary arteries as well. Coronary arteries were counted in 3-4 transverse cardiac sections, spaced 0.5 mm apart from the middle of the heart to the aortic sinus, and classified as either non-atherosclerotic, or atherosclerotic. Images of coronary arteries representing these categories are shown in Figures 3I–K. SR-B1 KO mice had, on average, ∼2 times as many atherosclerotic coronary arteries per heart section as both LDLR KO mice and ApoE KO mice (Figure 3L). As anticipated, all three KO strains developed significantly more coronary artery atherosclerosis than WT mice (Figure 3L).

### Blood cells and cytokines

**FIGURE 4:**
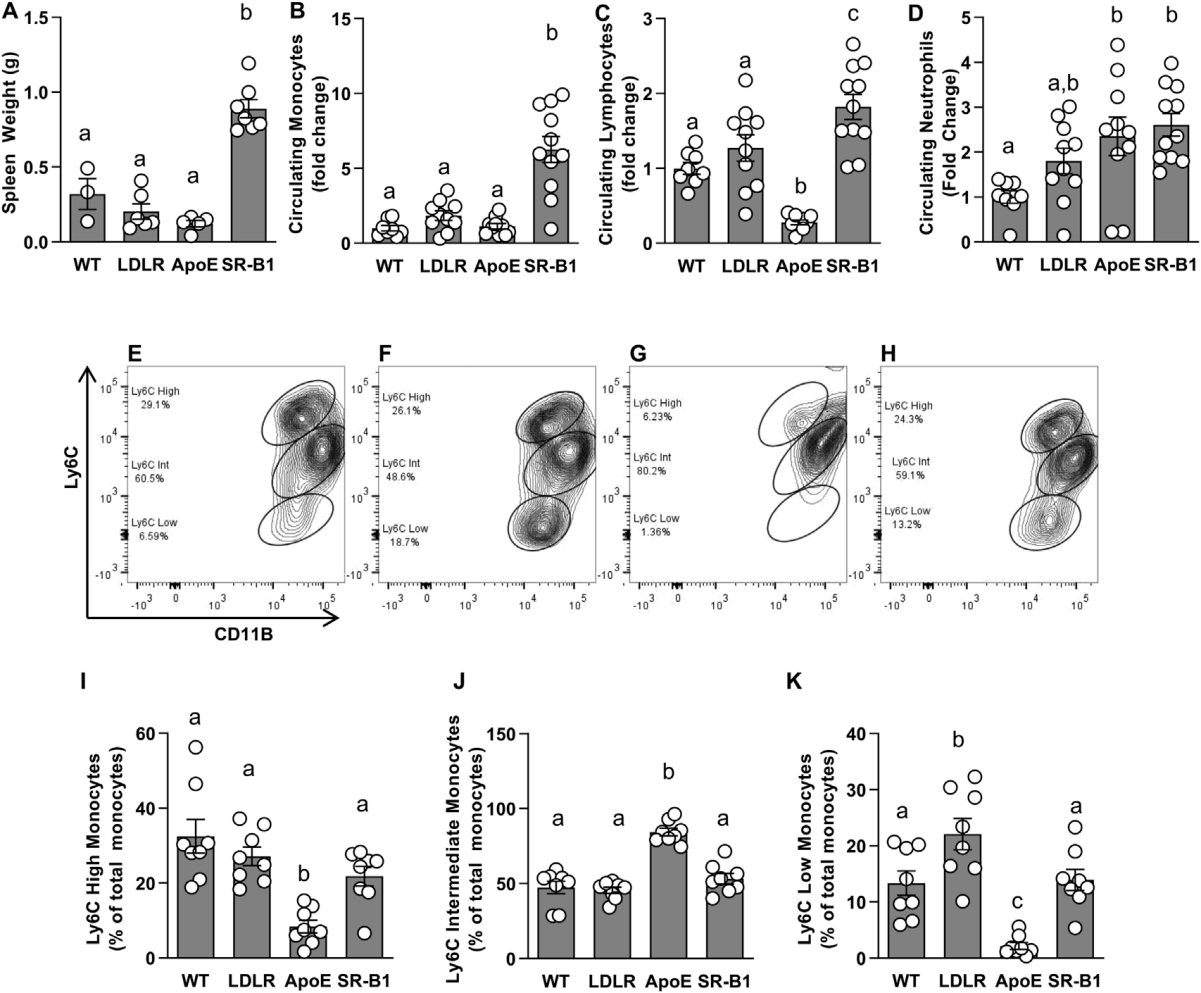
Circulating immune cells in HFCC-fed female WT, LDLR KO, ApoE KO and SR-B1 KO mice. **(A)** Spleens were collected and weighed after 20 weeks of HFCC feeding. Blood cells were analyzed from whole blood collected from the tail veins of mice after 6 weeks of HFCC feeding using a Hemavet Multi-species Cell Analyzer **(B–D)** or flow cytometry **(E–K)**. Average levels ± SEM of circulating monocytes **(B)** lymphocytes **(C)** and neutrophils **(D)** in each genotype group are shown, normalized to levels observed in WT mice (*N* = 8–11 per group). For flow cytometry, CD11b (shown) and CD115 double positive monocytes were analyzed for levels of Ly6C expression. Representative cytograms illustrating Ly6C expression patterns in monocytes C57B6 **(E)**, LDLR KO **(F)**, ApoE KO **(G)** and SR-B1 KO **(H)** mice. Flow cytometry data was analyzed using FloJo software. Graphs represent proportions of Ly6C High **(I)**, Ly6C Intermediate **(J)** and Ly6C Low **(K)** monocyte subsets in blood (*N* = 8 per group). Individual symbols represent samples from individual mice and bars with error bars represent average ± SEM of each group. The different letters indicate groups that are statistically significantly different from one another by one-way ANOVA with Tukey’s post-hoc test. **(A)** Overall ANOVA: *p* < 0.0001; multiple comparisons: *p* < 0.0001 for a vs. b; **(B)** Overall ANOVA: *p* = 0.0002; multiple comparisons: *p* < 0.001 for a vs. b; **(C)** Overall ANOVA: *p* < 0.0001; multiple comparisons: *p* < 0.005 for a vs. b; *p* < 0.03 for a vs. c; *p* < 0.0001 for b vs. c; **(D)** Overall ANOVA: *p* = 0.005; multiple comparisons: *p* < 0.025 for a *vs.* b; **(I)** Overall ANOVA: *p* < 0.0001; multiple comparisons: *p* < 0.02 for a vs. b; **(J)** Overall ANOVA: *p* < 0.0001; multiple comparisons: *p* < 0.0001 for a vs. b; **(K)** Overall ANOVA: *p* < 0.0001; multiple comparisons: *p* < 0.04 for a vs. b; *p* < 0.003 for b vs*.* c; *p* < 0.002 for a vs. c.

Given that HFCC-fed SR-B1 KO mice developed more extensive atherosclerosis than LDLR KO and ApoE KO mice fed the same diet despite having much lower plasma cholesterol levels, and that both immune cells and inflammation are major contributors to atherosclerosis, we analyzed circulating blood cell populations and levels of select plasma cytokines. We noticed that spleens from SR-B1 KO mice fed the HFCC diet were markedly larger than those from WT, LDLR KO or ApoE KO mice (Figure 4A). To analyze blood cells from HFCC-diet fed mice, blood was collected from tail veins of mice after 6 weeks of HFCC diet feeding. Blood cells were counted using a Hemavet multi species hematology analyzer. Red blood cells in chow-fed SR-B1 KO mice were slightly larger than those of WT, LDLR KO and ApoE KO mice, and platelet counts were significantly lower in chow-fed SR-B1 KO mice compared to WT and LDLR KO mice. However, when fed a HFCC diet for 6 weeks, SR-B1 KO mice exhibited substantially lower red blood cell counts and substantially larger red blood cells compared to WT, LDLR and ApoE KO mice (Supplementary Table S1). In chow-fed 10-week old mice, there were no significant differences in circulating neutrophils, lymphocytes or monocytes between any of the genotype groups aside from a slightly higher lymphocyte counts in LDLR KO mice compared to SR-B1 KO mice (data not shown). Strikingly, monocytes in HFCC-fed SR-B1 KO mice were ∼6 fold higher than observed in WT and ApoE KO mice, and ∼3-fold higher than in LDLR KO mice (Figure 4B). Lymphocytes in HFCC-fed SR-B1 KO mice were significantly elevated (∼1.8 fold compared to WT) while they were significantly lower in ApoE KO mice (Figure 4C). Neutrophils counts were elevated in HFCC diet-fed LDLR (∼1.8-fold) and ApoE and SR-B1 KO mice (∼2.5 fold) compared to HFCC diet-fed WT mice (Figure 4D). We also measured Ly6C expression levels in monocytes by flow cytometry (Figures 4E–K). SR-BI KO, LDLR KO and WT mice exhibited similar proportions of Ly6C^high^, Ly6C^int^ and Ly6C^low^ monocytes (Figures 4E–K). In contrast, ApoE KO mice mainly exhibited Ly6C^int^ monocytes in circulation, with much lower levels of Ly6C^high^ and Ly6C^low^ monocytes (Figures 4G, I–K). By virtue of the higher numbers of circulating monocytes in SR-B1 KO mice fed the HFCC diet (Figure 4B), the absolute numbers of circulating Ly6C^high^ monocytes are higher in SR-BI KO mice, than in LDLR KO, ApoE KO or control WT mice.

**FIGURE 5:**
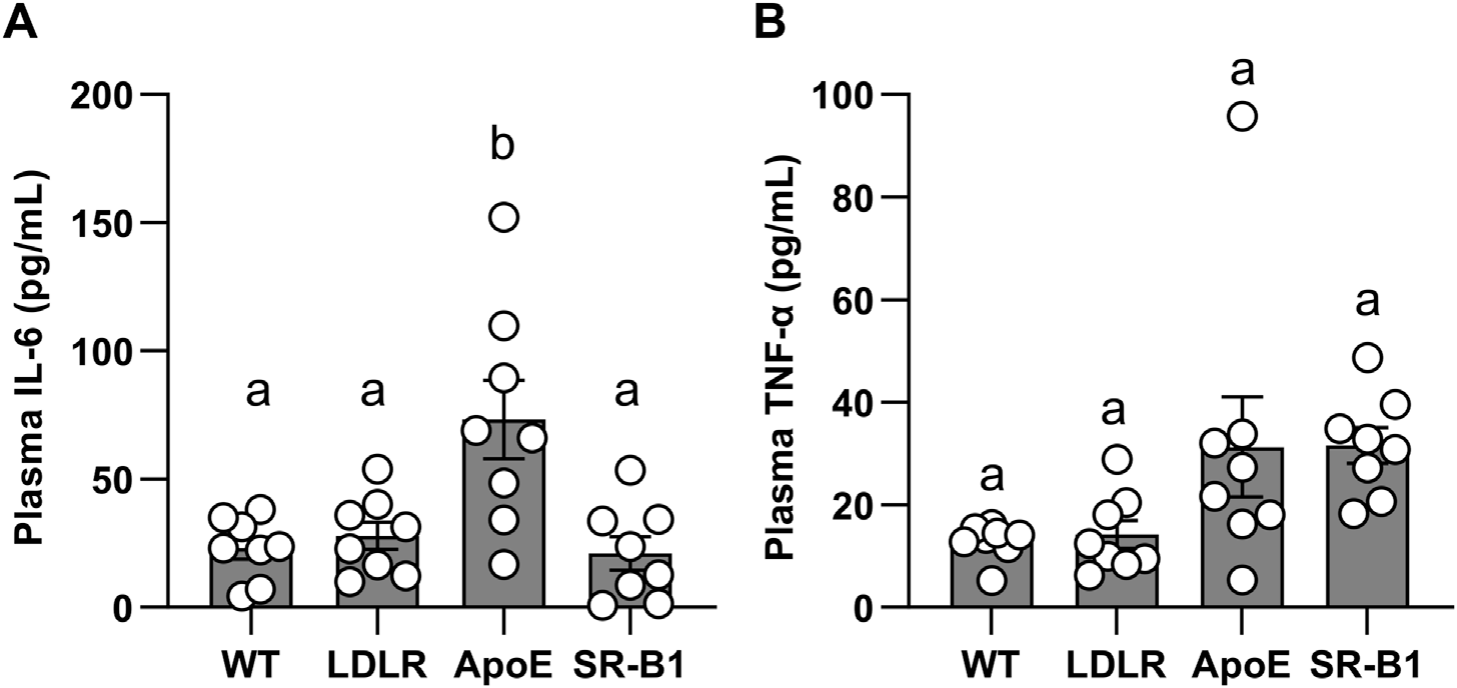
Levels of circulating IL-6 and TNF-α in plasma. IL-6 **(A)** and TNF-α **(B)** were measured by ELISA in plasma from female mice after 20 weeks of HFCC feeding. Individual symbols represent samples from individual mice and bars with error bars represent average ± SEM of *N* = 8 per group. Different letters indicate statistically significant differences from one another by one-way ANOVA with Tukey’s post-hoc test. **(A)** Overall ANOVA: *p* = 0.0008; multiple comparisons: *p* < 0.01 for a vs. b. **(B)** Overall ANOVA: *p* = 0.02; multiple comparisons: *p* > 0.05 (not statistically significant) for each comparison.

IL-6 and TNF-α levels in all groups of mice were below the limit of detection of the assay prior to the onset of HFCC feeding. After 20 weeks of HFCC diet-feeding, IL-6 levels in ApoE KO mice were significantly elevated compared WT, LDLR and SR-B1 KO mice, which all had similar IL-6 levels (Figure 5A). Plasma TNF-α concentrations (Figure 5B) showed trends to higher levels in ApoE KO and SR-B1 KO mice compared to WT and LDLR KO mice, but differences did not reach statistical significance upon multiple comparisons test. Plasma corticosterone levels were analyzed in separate cohorts of female WT and SR-B1 KO mice fed HFCC diet (for 12 weeks) that had been fasted for 14 h (to induce stress) prior to blood collection. Under these conditions, the SR-B1 KO mice exhibited lower plasma concentrations of corticosterone than the WT mice (Supplementary Figure S2), consistent with previous reports (Hoekstra et al., 2008; Hoekstra et al., 2009; Hoekstra et al., 2013; Reece et al., 2021).

### Coronary artery VCAM-1 levels

**FIGURE 6:**
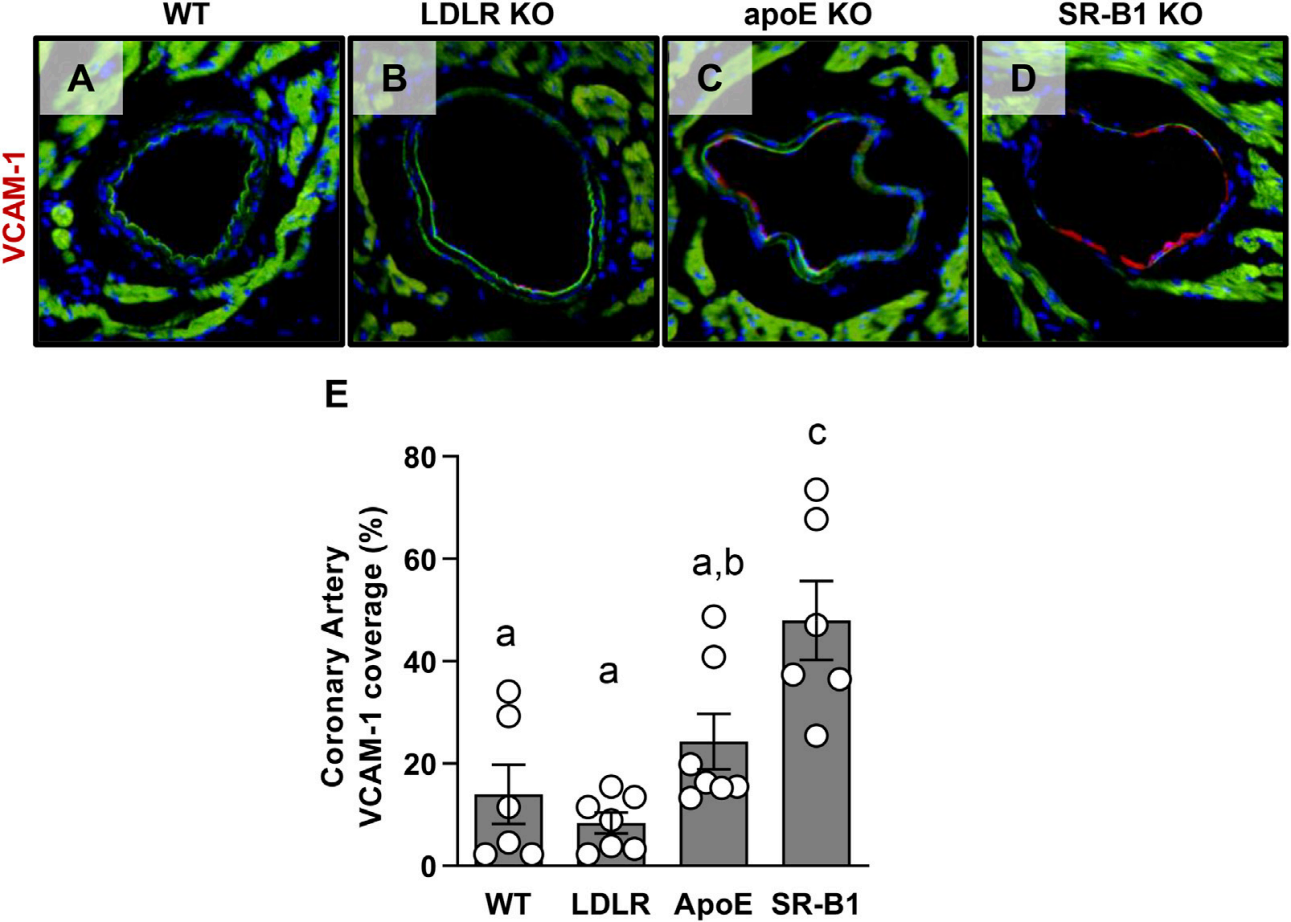
Expression of VCAM-1 in coronary artery endothelial cells in HFCC-fed female WT, LDLR KO, ApoE KO and SR-B1 KO mice. Coronary arteries in transverse cryosections of myocardium were stained for VCAM-1 expression by immunofluorescence. Representative images of VCAM-1-stained coronary arteries from mice from each indicated genotype group are shown in **(A–D)**. The length VCAM-1 positive staining in coronary artery endothelium was measured using ImageJ software and expressed as a percentage of total artery endothelium length (the cumulative circumference of each artery counted). **(E)** VCAM-1 for 6-7 mice per group. Individual symbols represent samples from individual mice and bars with error bars represent average ± SEM. Different letters indicate statistically significant differences from one another by one-way ANOVA with Tukey’s post-hoc test. Overall ANOVA: *p* = 0.0002; multiple comparisons: *p* < 0.002 a vs. c; *p* = 0.026 b vs. c.

We have previously shown that SR-B1/LDLR double KO mice that are susceptible to severe occlusive coronary artery atherosclerosis have high levels of adhesion molecule expression in their coronary arteries when compared to LDLR single KO controls. Since VCAM-1 expression is thought to precede the development of atherosclerosis and may be a major factor in determining where atherosclerotic plaques develop in the vasculature, we measured VCAM-1 expression in the coronary arteries of the mice in this study. Figures 6A–D shows representative images of VCAM-1 immunofluorescence in coronary arteries from WT, LDLR KO, ApoE KO and SR-B1 KO mice. As a proportion of total endothelium analyzed, SR-B1 KO mice expressed significantly more VCAM-1 in coronary artery endothelium compared to all other groups (Figure 6E), suggesting that SR-B1 plays a role in suppressing VCAM-1 expression in coronary arteries.

## Discussion

In mice that are genetically susceptible to experimental atherosclerosis, SR-B1 deficiency results in the development of severe occlusive coronary artery atherosclerosis, myocardial infarction and early death (Braun et al., 2002; Zhang et al., 2005; Fuller et al., 2014). This phenotype is associated with monocytosis, inflammation and elevated adhesion molecule expression in the coronary endothelium (Fuller et al., 2014). SR-B1 single KO mice develop extensive atherosclerosis in response to HFCC diet-feeding (Harder et al., 2007); however, HFCC diet-fed SR-B1 KO mice are not a commonly used model of experimental atherosclerosis and have not been comprehensively characterized. In this study, we compared SR-B1 KO mice fed the HFCC diet for 20 weeks to WT, LDLR KO and ApoE KO mice fed the same diet for the same duration. We show that SR-B1 KO mice developed large atherosclerotic plaques in the aortic sinus, which in females (Figure 1) were similar in size, but in males (Supplementary Figure S1) were on average half the size of those in similarly treated LDLR KO and ApoE KO mice. In the descending aorta and coronary arteries, female SR-B1 KO mice developed more extensive atherosclerosis than LDLR KO and ApoE KO mice. This was associated with leukocytosis, particularly monocytosis, and elevated VCAM-1 expression in coronary arteries and despite much lower plasma cholesterol levels in SR-B1 KO mice.

While hypercholesterolemia is a major risk factor for atherosclerosis, the results of this study highlight the requirement for other factors that facilitate atherosclerotic plaque development. The finding that total plasma cholesterol in HFCC fed SR-B1 KO mice, while elevated compared to chow fed SR-B1 KO mice, is not significantly different from HFCC fed WT mice, indicates that disease development in these mice was driven by factors other than simply elevated cholesterol induced by the HFCC diet. SR-B1 KO mice have elevated free cholesterol: total cholesterol ratios compared to WT, LDLR KO and ApoE KO mice. This indicates a compositional difference in lipoproteins in SR-B1 deficient mice that may lead to defects in normal lipoprotein function. SR-B1 KO mice also have a major defect in reverse cholesterol transport, leading to large HDL particles that are unable to off-load cholesterol to the liver (Trigatti et al., 2003). To what extent this might influence the capacity of HDL to accept cholesterol from peripheral tissues, impact retention of cholesterol in macrophages in the plaque, and/or influence the ability of HDL to induce atheroprotective intracellular signaling pathways, especially those that are dependent on SR-B1 remains unclear and requires further investigation.

In addition to elevated cholesterol, inflammation and the immune system also play a significant role in determining the progression of atherosclerosis (Libby, 2002; Libby, 2021; Soehnlein and Libby, 2021). High levels of systemic pro-inflammatory cytokines can activate endothelial cells as well as immune cells in the atherosclerotic plaque and perpetuate atherosclerosis. Moreover, leukocytosis is a documented risk factor for atherosclerosis in humans and animal models (Tall et al., 2012). We measured IL-6 and TNF-α levels in the plasma and found that TNF-α trended towards elevated levels in ApoE KO and SR-B1 KO mice compared to LDLR KO and WT mice, but this did not reach statistical significance. SR-B1 KO mice exhibited substantial leukocytosis, with monocytes elevated up to 6-fold compared to other groups in the study. Although the proportion of circulating monocytes with high levels of Ly6C cell surface expression were not higher in SR-BI KO mice, the absolute numbers of Ly6C^high^ monocytes in circulation were higher by virtue of the overall increased numbers of circulating monocytes. This, combined with the increased VCAM-1 expression, at least in coronary arteries, might explain the increased susceptibility of these arteries to atherosclerosis in SR-BI KO mice compared to the LDLR and ApoE KO mice. These data suggest that while cholesterol is not elevated to the same extent as in LDLR and ApoE KO mice, it is sufficiently high in SR-B1 KO mice to facilitate atherosclerotic plaque formation under conditions of extensive monocytosis and elevated inflammation.

Elevated inflammatory markers in the plasma and leukocytosis are systemic risk factors that would be expected to influence overall atherosclerosis, but would not be expected to impact the distribution of atherosclerosis in different regions of the vasculature. One of the interesting findings in this study is the different distribution of atherosclerosis observed in SR-B1 KO mice compared to the other groups. Like LDLR KO and ApoE KO mice, SR-B1 KO mice developed extensive atherosclerosis in normally susceptible arterial sites such as the aortic sinus and aortic arch; however, under the feeding conditions used, female SR-B1 KO mice also developed higher levels of atherosclerosis than corresponding ApoE or LDLR KO mice along the entire length of their descending aortas as well as in coronary arteries. This may be the consequence of the absence of hepatic SR-B1 expression and subsequent alterations in HDL structure/function affecting the vasculature. Alternatively, it is possible that the absence of SR-B1 in the endothelial cells of the arteries themselves may enhance the initiation of atherosclerosis. It is well documented that atherosclerosis in mice tends to develop at sites along the vasculature that experience non-laminar blood flow (Hajra et al., 2000; Won et al., 2007). Endothelial cells in these regions experience low shear stress and turbulent blood flow and tend to be more permeable to components of the blood, including circulating lipoproteins (Chatzizisis et al., 2007). These regions also have up-regulated expression of inflammatory genes including adhesion molecules, creating favorable sites for monocytes to attach to the artery wall (Iiyama et al., 1999; Hajra et al., 2000; Won et al., 2007). The expression of these adhesion molecules is thought to be a strong predictor of locations that are likely to develop atherosclerotic plaques (Iiyama et al., 1999). HDL has been shown to protect endothelial cells against apoptosis induced by multiple stimuli (Suc et al., 1997; Sugano et al., 2000; Nofer et al., 2001). HDL also enhances the activity of endothelial nitric oxide synthase (eNOS) in endothelial cells in an SR-B1 dependent manner (Yuhanna et al., 2001; Seetharam et al., 2006; Mineo and Shaul, 2013). This results in eNOS-dependent suppression of TNF-α-induced adhesion molecule expression (Kimura et al., 2006). HDL binding to SR-B1 also induces eNOS-independent EC migration (Seetharam et al., 2006), which may influence endothelial repair. On the other hand, others have demonstrated that SR-B1 mediates transcytosis of LDL across endothelial cells from the lumen to the sub-endothelial space of arteries and that endothelial selective KO of SR-B1 protected mice against atherosclerosis development (Armstrong et al., 2015; Ghaffari et al., 2018; Huang et al., 2019; Ghaffari et al., 2021). Despite this, we observe, consistent with other reports, that whole body SR-B1 KO increased susceptibility of mice to atherosclerosis development, in this case triggered by the HFCC diet, and that the levels of atherosclerosis, at least under the conditions (diet and feeding time) employed herein, were comparable to those observed in ApoE KO and LDLR KO mice. This suggests that any detrimental effects of knocking out SR-B1 in tissues/cell types other than endothelial cells outweigh any beneficial effects of reduced trans-endothelial transport of atherogenic lipoproteins resulting from the absence of SR-B1 expression in endothelial cells. For example, we demonstrate that similar to SR-B1/LDLR dKO mice (Fuller et al., 2014), coronary arteries of SR-B1 single KO mice exhibit higher levels of VCAM-1 compared to the wild type, ApoE KO or LDLR KO mice analyzed in this study. These data support the notion that SR-B1-mediated protection against the development of atherosclerosis in mouse arteries may include its role in suppressing VCAM-1 expression in endothelial cells.

Alternatively, it is possible that the increased VCAM-1 levels seen in coronary arteries of SR-B1 KO mice may be the result of other factors, such as the influence of abnormal circulating lipoproteins resulting from the absence of SR-B1 in the liver. The influence of lipoproteins from SR-B1 KO mice on endothelial function requires further investigation. Another factor may be the influence of a lack of SR-B1 on stress-induced corticosterone production. Endogenous corticosteroids are known to suppress VCAM-1 levels in endothelial and other cells (Ihm et al., 1996; Atsuta et al., 1999; Cavalcanti et al., 2007). SR-B1 is required for cholesterol uptake from HDL by adrenocortical cells and, in SR-B1 KO mice, cells in the adrenal cortex and other steroidogenic tissues are depleted of endogenous cholesterol ester stores (Rigotti et al., 1997; Temel et al., 1997; Trigatti et al., 2003). As a result of the absence of SR-B1 expression in adrenocortical cells in SR-B1 KO mice, they exhibit reduced plasma corticosterone levels (Supplementary Figure S2) due to an impaired ability to induce corticosterone production and release under conditions of stress, such as fasting (Hoekstra et al., 2008; Hoekstra et al., 2009; Hoekstra et al., 2013; Reece et al., 2021). In contrast, neither LDLR nor ApoE deficiency reportedly impairs stress-induced corticosterone production by adrenal glands (Kraemer et al., 2007; van der Sluis et al., 2015; Hoekstra and Van Eck, 2016; van der Sluis et al., 2020). Thus, the reduced stress-induced corticosterone levels in SR-B1 KO mice may also be a factor that contributes to the increased levels of VCAM1 in coronary arteries. Whether other arteries also exhibit increased VCAM-1 levels, and the precise mechanisms underlying the increased VCAM-1, however, remain to be determined.

In conclusion, HFCC fed SR-B1 KO mice develop widespread atherosclerosis in multiple arteries. We believe this phenotype is facilitated by slightly elevated cholesterol from the diet, but driven by a multitude of cholesterol independent factors such as inflammation, dysregulation of the haematopoietic system, and enhanced activation of vascular endothelial cells. Given that SR-B1 single KO mice possess intact mechanisms for LDL and VLDL clearance from the blood stream, they may prove a useful model of experimental atherosclerosis that is suitable for testing therapeutic agents, such as statins and PCSK9 inhibitors, which rely on the enhancement of this system as a mechanism for cholesterol lowering.

## Data Availability

The original contributions presented in the study are included in the article/Supplementary Material, further inquiries can be directed to the corresponding author.
