# Supplementary material for: Extensive diet-induced atherosclerosis in scavenger receptor class B type 1-deficient mice is associated with substantial leukocytosis and elevated vascular cell adhesion molecule-1 expression in coronary artery endothelium
Source: Front Physiol. 2023 Jan 12;13:1023397. doi: 10.3389/fphys.2022.1023397 (PMC9877335; doi:10.3389/fphys.2022.1023397)
Supplement: Supplementary file 1 [file DataSheet1.PDF]

Supplementary Material

1 Supplementary Data

1.1 Supplementary Figure 1

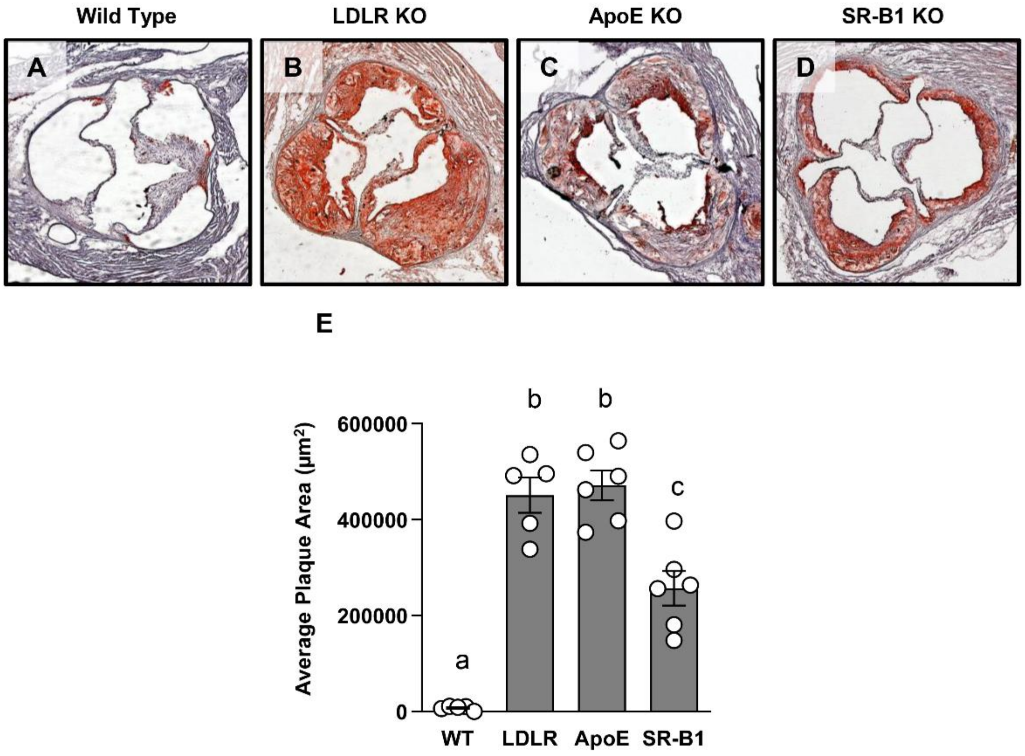

Supplementary Figure 1– Atherosclerosis in the aortic sinus of male wild type, LDLR KO, ApoE KO and SR-B1 KO mice fed the HFCC for 20 weeks. Male mice were fed the HFCC diet for 20 weeks starting at 10 weeks of age. Representative oil red O-stained transverse cryosections of the aortic sinus are shown in panels A-D, quantification of average plaque size in the aortic sinus is shown in E. Individual symbols represent data from individual mice and bars with error bars represent average ± SEM of each group (N=5-6/group). Bars with different letters are statistically significantly different from one another by one-way ANOVA with Tukey's post-hoc test. P values are: Overall ANOVA: $P<0.0001$; multiple comparisons: $P<0.0001$ for a vs b; $P<0.002$ for a or b vs c.

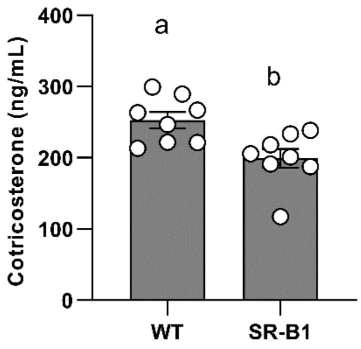

Supplementary Figure 2– Effects of SR-B1 deficiency on corticosterone levels in female mice. Female wild type (WT) or SR-B1 KO mice were fed the HFCC diet for 12 weeks, fasted and plasma corticosterone levels were determined by ELISA. Data points represent individual mice (n=8/group). Bars represent averages and error bars represent SEM. Different letters indicate statistical significance between groups. P=0.008 by Mann-Whitney Rank Sum test.

1.2 Supplementary Table

Supplementary Table. Hematology profiles for wild type, LDLR KO, apoE KO and SR-B1 KO mice fed either chow or HFCC diet for 20 weeks. Whole blood was collected from tail or submandibular veins of female 10-12 week old chow-fed mice or mice fed the HFCC diet for 20 weeks. Hematology profiles were generated using a Hemavet Multi-Species Hematology System (Drew Scientific). Results shown are mean ± SEM for each output. Sample sizes are indicated in parentheses in the genotype column. Values with different letters are statistically significantly different from each other by one-way ANOVA with Tukey's post-hoc test within each diet group.

Genotype	Diet	RBC Count (M/μl)	Hematocrit (%)	Mean RBC Volume (fL)	RBC Dist. Width (%)	Platelet Count (K/μl)
Wild Type	Chow (6)	10.19 ± 0.08 [a]	45.7 ± 0.8 [a]	44.8 ± 0.5 [a]	16.63 ± 0.41 [a]	743 ± 50 [a]
	HFCC (8)	9.92 ± 0.41 [a]	44.2 ± 2.1 [a]	44.5 ± 0.5 [a,b]	17.3 ± 0.4 [a]	754 ± 97 [a]
LDLR KO	Chow (8)	10.81 ± 0.16 [a,b]	44.5 ± 0.7 [a]	45.4 ± 0.1 [a]	15.94 ± 0.13 [a]	730 ± 48 [a]
	HFCC (10)	10.11 ± 0..55[a]	45.6 ± 2.8 [a]	45.0 ± 0.4 [b]	16.5 ± 0.1 [a]	701 ± 96 [a]
apoE KO	Chow (9)	9.40 ± 0.13 [b]	43.6 ± 1.0 [a]	46.4 ± 0.6 [a,b]	16.78 ± 0.22 [a]	625 ± 67 [a,b]
	HFCC (10)	9.63 ± 0.33 [a]	41.2 ± 1.4 [a]	42.8 ± 0.3 [a]	18.0 ± 0.1 [a]	792 ± 132 [a]
SR-B1 KO	Chow (9)	9.61 ± 0.15 [b]	45.6 ± 0..7 [a]	47.4 ± 0.3 [b]	18.21 ± 0.24 [b]	453 ± 41 [b]
	HFCC (11)	5.10 ± 0.33 [b]	41.0 ± 2.7 [a]	80.5 ± 0.6 [d]	22.1 ± 0.6 [b]	570 ± 46 [a]
